# Construction and clinical validation of benign paroxysmal positional vertigo intelligent auxiliary diagnosis model based on big data analysis

**DOI:** 10.3389/fneur.2025.1636696

**Published:** 2025-08-07

**Authors:** Jingyang Han, Tao Wang, Xiaoyan Du, Yali Wang, Ziyang Guo, Dandan Li, Xinjun Yu

**Affiliations:** ^1^Geriatric Medicine Department, Affiliated Hospital of Shandong Second Medical University, Weifang, China; ^2^School of Clinical Medicine, Affiliated Hospital of Shandong Second Medical University, Weifang, China

**Keywords:** benign paroxysmal positional vertigo, intelligent auxiliary diagnosis, big data, machine learning, SRM-IV Vertigo diagnosis and treatment system

## Abstract

**Background:**

Benign paroxysmal positional vertigo (BPPV) is the most common type of vertigo in clinical practice. Previous studies have suggested that inflammatory responses and metabolic disorders may be involved in the pathogenesis of BPPV, but systematic analyses based on large samples are lacking. The aim of this study is to construct an intelligent auxiliary diagnostic model for BPPV based on the big data of SRM-IV vertigo diagnostic and treatment system, and to carry out clinical validation.

**Methods:**

The clinical data of 522 vertigo patients were retrospectively analyzed, including 303 BPPV patients and 219 non-BPPV patients. LASSO regression and random forest algorithm were used to screen feature variables, and based on the screened feature variables, multifactor logistic regression analysis was performed to establish a prediction model for BPPV auxiliary diagnosis. Finally, the model was applied to BPPV patients diagnosed by SRM-IV diagnosis and treatment system for external validation.

**Results:**

Multifactorial logistic regression analysis showed that disease duration, neutrophils, lymphocytes, C-reactive protein (CRP), ferritin, and vitamin D deficiency were independent risk factors for the diagnosis of BPPV (OR>1, *p* < 0.05), monocyte count was an independent protective factor for the diagnosis of BPPV (OR<1, *p* < 0.05), and the area under curve (AUC) was 0.927.

**Conclusion:**

The intelligent assisted diagnostic model of BPPV constructed based on the big data of SRM-IV vertigo diagnostic and treatment system has high diagnostic accuracy and clinical application value, and it is expected to assist the clinicians to improve the diagnostic efficiency.

## 1 Introduction

Vertigo disorders are very common in clinical practice, with peripheral vertigo being the most common, accounting for about 70% of all vertigo cases. Specific disorders include BPPV, Meniere’s disease, vestibular neuroinflammation and vestibular paroxysm (1). Among these disorders, BPPV has a high prevalence, accounting for about more than 1/3 of all vertigo cases (2, 3). The pathogenic mechanisms of vertigo are complex, and the related clinical research faces great challenges. Even experienced physicians are often troubled by the diagnosis of the primary disease when seeing patients with vertigo.

In recent years, with the advancement of algorithmic science and the expansion of research ideas, AI diagnosis of vertigo-related diseases tends to look for details that are not easily found by humans in diagnostic and therapeutic work through AI techniques (4, 5). Some researchers used artificial intelligence analysis to develop the optimal operation plan of the smart swivel chair reset system to provide patients with the best solution and optimal experience of reset, which is an innovative attempt to integrate artificial intelligence with traditional reset methods (6). Baydan-Aran et al. (7) used the random forest algorithm to construct a diagnostic model for BPPV based on clinical features, with an AUC of 0.89. Mun et al. (8) developed an automated BPPV diagnosis system by combining deep learning and nystagmus video analysis with an accuracy of 91.2%. However, these studies were mainly based on clinical symptoms and signs, and less frequently incorporated laboratory biomarkers for comprehensive analysis.

The SRM-IV diagnosis and treatment system is currently one of the devices for the treatment of canalith ectopia, motion sickness (motion sickness, seasickness, airsickness), and acrophobia at home and abroad (9, 10). This diagnostic and treatment system can not only carry out the diagnosis of vertigo and screening of dizziness patients, but also test the vestibular function. It has a good effect on fixing the patient to the seat, rotating the seat, monitoring nystagmus, and accurately locating the area where the canalith is located, and formulating the most effective reduction plan, especially for patients with refractory BPPV and atypical BPPV. The technical advantages of the SRM-IV diagnosis and treatment system are mainly as follows: quantifying nystagmus parameters and automatically generating nystagmus curves; precise positioning of canalith semicircular canals; built-in reset scheme algorithm. Compared with traditional manual reduction, SRM-IV significantly improves the diagnostic efficiency of refractory BPPV (eg, involving multiple semicircular canals) (sensitivity 92.3%, specificity 89.7%) (11). While other swivel chair systems, such as the TRV Chair, rely primarily on manually operated nystagmus observations, the SRM-IV automates data analysis throughout the process.

With the development of computer technology, the effective integration of Artificial Intelligence (AI) is expected to improve the diagnosis and treatment of clinical diseases, enhance the efficiency of clinicians, and then alleviate the strain on medical resources. Some researchers have used AI analysis to formulate the optimal operation plan of the intelligent swivel chair reset system to provide patients with the best solution and optimal experience of reset, which is an innovative attempt to integrate AI with traditional reset methods.

In this study, we planned to construct an intelligent auxiliary diagnosis model of BPPV based on the SRM-IV vertigo diagnosis and treatment system based on big data analysis. Through big data analysis and machine learning methods, key characteristic variables for BPPV diagnosis were screened and multifactorial logistic regression models were constructed, aiming to provide a convenient and fast diagnostic tool for clinical diagnosis of BPPV. This study hypothesizes that inflammatory responses and metabolic disorders are involved in the pathogenesis of BPPV. By analyzing changes in relevant biomarkers, we aim to identify high-risk populations for BPPV. The research objective is to establish a BPPV risk assessment model based on inflammatory markers and metabolic indicators.

## 2 Methods

### 2.1 Study population and data collection

This study was a retrospective case analysis and included 522 patients with vertigo diagnosed in the SRM-IV vertigo diagnostic and treatment system. Patient data were derived from the system’s big data platform and spanned a 1-year period (January 2024–January 2025). The study was approved by the Ethics Committee of the Affiliated Hospital of Shandong Second Medical University. The inclusion criteria were (1) meeting the diagnostic criteria for vertigo in the Vertigo Diagnostic and Treatment Guidelines, and (2) having a complete clinical history, including demographic information (gender, age, marital status, occupation, and level of education), medical history (duration of the disease, side of the onset of the disease, comorbidities, etc.), laboratory indices (white blood cell count, neutrophil count, lymphocyte count, monocyte count, C-reactive protein, ferritin, IL6) and the degree of hearing impairment. The exclusion criteria were (1) incomplete clinical data or missing key information, and (2) the presence of other definite etiological factors that could lead to vertigo, such as central nervous system diseases and drug intoxication. Based on the diagnosis, patients were divided into BPPV group (n = 303) and non-BPPV group (n = 219). All data were desensitized to protect patient privacy.

### 2.2 Feature variable screening and machine learning

To identify the key feature variables for BPPV diagnosis, the following machine learning methods were used in this study:(1) LASSO regression analysis: The LASSO regression model was applied for feature selection. The optimal penalty coefficient *λ* is determined through cross-validation and the corresponding feature variable at Lambda.1se is selected. (2) Random Forest: The randomForest package was used, setting ntree = 1,000 and mtry = 3 (square root of the number of predictor variables). The importance of the variables was assessed using both Gini coefficient decline (type1) and replacement importance (type2) methods, and the top 10 ranked variables were selected. To avoid overfitting, out-of-bag (OOB) errors were used to assess model performance.

### 2.3 Multifactor logistic regression analysis and predictive model construction

Multifactor Logistic regression analysis was performed based on the feature variables screened by machine learning. Whether the diagnosis of BPPV was made was used as the dependent variable, and the above feature variables were included in the model as independent variables. Statistically significant variables were screened using stepwise regression and the respective odds ratios (OR) and 95% confidence intervals (CI) were calculated. Based on the results of the multifactorial logistic regression analysis, a predictive model for BPPV-assisted diagnosis was constructed. The model was presented in the form of a column-line graph for easy clinical application.

### 2.4 Validation of predictive models and evaluation of clinical applications

The established prediction model was applied to another batch of BPPV patients diagnosed in the SRM-IV vertigo diagnosis and treatment system (validation set), and the prediction effect of the model on an independent dataset was evaluated. The validation set was an independent cohort (n = 112) of patients from subsequent visits to the same center. (1) Receiver Operating Characteristic (ROC) Curve and AUC: ROC curves were plotted and AUC was calculated to assess the discriminatory power of the model. The higher the AUC value, the higher the prediction accuracy of the model. (2) Clinical calibration curves: The calibration of the model is assessed by plotting clinical calibration curves comparing the difference between the predicted probability and the actual incidence. The closer the calibration curve is to the diagonal, the better the model’s predictions match the actual situation. (3) Clinical Decision Curve and Clinical Impact Curve: Draw the clinical decision curve and clinical impact curve to evaluate the clinical net benefit rate and patient benefit of the model under different threshold probabilities to evaluate the clinical application value of the model.

### 2.5 Statistical analysis

SPSS 26.0 and R 4.1.0 software were used for statistical analysis. Normally distributed continuous data were expressed as mean ± standard deviation (Mean ± SD), and T-test was used for comparison between groups. Non-normally distributed continuous data were presented as median (interquartile range, Median [Q1, Q3]) and Mann–Whitney U test was used for comparison between groups. Numerological data were expressed as the number of cases [percentage, n (%)] and comparisons between groups were performed using either the chi-square test or Fisher’s exact test. *p* < 0.05 was statistically significant.

## 3 Results

### 3.1 Comparative analysis of the proportional risk of BPPV occurrence and baseline data in vertigo patients

A total of 522 subjects were included in this study, of which 303 were diagnosed with BPPV and 219 were non-BPPV. There was no significant difference in gender composition between the two groups (*p* = 0.9137; Table 1), but there was a significant difference in age distribution (*p* < 0.0001), and the mean age of the BPPV group was higher than that of the non-BPPV group (57.7 ± 19.0 vs. 50.8 ± 14.6). There was also a significant difference between the two groups in terms of disease duration (*p* < 0.0001), with a median disease duration of 4.88 days [0.75; 9.33] in the BPPV group and 7.87 days [0.49; 14.8] in the non-BPPV group.

**Table 1:** Comparative analysis of the proportional risk of BPPV occurrence and baseline data in vertigo patients.

General characteristics	All	Non-BPPV	BPPV	*p* overall
	*N* = 522	*N* = 303	*N* = 219	
Gender				0.9137
Female	281 (53.8%)	162 (53.5%)	119 (54.3%)	
Male	241 (46.2%)	141 (46.5%)	100 (45.7%)	
Age	54.8 ± 17.6	57.7 ± 19.0	50.8 ± 14.6	<0.0001
Disease duration	5.63 [0.49;14.8]	4.88 [0.75;9.33]	7.87 [0.49;14.8]	<0.0001
Marital status				0.2836
Unmarried	128 (24.5%)	80 (26.4%)	48 (21.9%)	
Married	394 (75.5%)	223 (73.6%)	171 (78.1%)	
Occupation				0.8054
Employees	223 (42.7%)	126 (41.6%)	97 (44.3%)	
Workers	191 (36.6%)	114 (37.6%)	77 (35.2%)	
Other	108 (20.7%)	63 (20.8%)	45 (20.5%)	
Education level				0.1031
High school	119 (22.8%)	67 (22.1%)	52 (23.7%)	
Junior high school	120 (23.0%)	77 (25.4%)	43 (19.6%)	
Primary school	188 (36.0%)	98 (32.3%)	90 (41.1%)	
University and above	95 (18.2%)	61 (20.1%)	34 (15.5%)	

In terms of laboratory indicators, there was no significant difference in white blood cell count between the two groups (*p* = 0.1948), but there were significant differences in neutrophil (*p* = 0.0095), lymphocyte (*p* = 0.0182), monocyte (*p* = 0.0001), CRP (*p* < 0.0001), ferritin (*p* = 0.0023), and IL-6 (*p* < 0.0001). The levels of monocytes in the BPPV group were higher than those in the non-BPPV group, while the levels of CRP, ferritin, and IL-6 were lower than those in the non-BPPV group (Table 2).

**Table 2:** Clinical and laboratory characteristics of patients with vertigo.

Clinical and laboratory characteristics	All	Non-BPPV	BPPV	*p* overall
	*N* = 522	*N* = 303	*N* = 219	
White blood cell	7.72 [0.48;18.2]	7.72 [3.37;12.3]	7.83 [0.48;18.2]	0.1948
Neutrophil	5.69 [1.32;12.1]	5.60 [1.68;8.29]	5.81 [1.32;12.1]	0.0095
Lymphocyte	2.24 [0.39;3.76]	2.19 [0.39;3.76]	2.27 [1.28;3.66]	0.0182
Monocyte	1.27 [0.55;2.09]	1.38 [0.55;2.09]	1.18 [0.73;1.74]	<0.0001
CRP	24.9 [1.64;45.7]	20.9 [1.64;42.2]	28.9 [15.8;45.7]	<0.0001
Ferritin	42.9 [−35.13;119]	40.3 [−7.94;96.5]	48.0 [−35.13;119]	0.0023
IL-6	9.66 [0.71;20.3]	8.99 [0.99;19.0]	10.2 [0.71;20.3]	<0.0001
Hearing loss				0.4563
Mild	323 (61.9%)	189 (62.4%)	134 (61.2%)	
Moderate	119 (22.8%)	64 (21.1%)	55 (25.1%)	
Severe	80 (15.3%)	50 (16.5%)	30 (13.7%)	
Side of onset				0.3679
Bilateral	105 (20.1%)	58 (19.1%)	47 (21.5%)	
Left	202 (38.7%)	125 (41.3%)	77 (35.2%)	
Right	215 (41.2%)	120 (39.6%)	95 (43.4%)	
Anxiety	68 (13.0%)	23 (7.59%)	45 (20.5%)	<0.0001
Depression	51 (9.77%)	12 (3.96%)	39 (17.8%)	<0.0001
Insomnia	108 (20.7%)	58 (19.1%)	50 (22.8%)	0.3590
Smoking	118 (22.6%)	60 (19.8%)	58 (26.5%)	0.0900
Drinking	64 (12.3%)	35 (11.6%)	29 (13.2%)	0.6556
Essential hypertension	79 (15.1%)	38 (12.5%)	41 (18.7%)	0.0687
Vitamin D deficiency	224 (42.9%)	79 (26.1%)	145 (66.2%)	<0.0001
Stroke	71 (13.6%)	32 (10.6%)	39 (17.8%)	0.0242
Diabetes mellitus	64 (12.3%)	29 (9.57%)	35 (16.0%)	0.0386
Hyperuricemia	71 (13.6%)	32 (10.6%)	39 (17.8%)	0.0242
Vestibular migraine	82 (15.7%)	40 (13.2%)	42 (19.2%)	0.0836
Traumatic head injury	40 (7.66%)	19 (6.27%)	21 (9.59%)	0.2150
Head trauma	50 (9.58%)	21 (6.93%)	29 (13.2%)	0.0234
Tympanitis	99 (19.0%)	40 (13.2%)	59 (26.9%)	0.0001

There was no significant difference in the degree of hearing loss between the two groups (*p* = 0.4563). There were also no significant differences in incidence (*p* = 0.3679), marital status (*p* = 0.2836), occupation (*p* = 0.8054), and education level (*p* = 0.1031) between the two groups. In terms of comorbidities, anxiety (*p* < 0.0001) and depression (*p* < 0.0001) were significantly different between the two groups, and the incidence of anxiety and depression was higher in the non-BPPV group than in the BPPV group. Vitamin D deficiency was more common in the non-BPPV group (*p* < 0.0001). Stroke (*p* = 0.0242), diabetes mellitus (*p* = 0.0386), hyperuricemia (*p* = 0.0242), and history of head surgery (*p* = 0.0234) were significantly different between the two groups, and were more common in the non-BPPV group. There were no significant differences in essential hypertension (*p* = 0.0687), vestibular migraine (*p* = 0.0836), head trauma (*p* = 0.2150), insomnia (*p* = 0.3590), smoking (*p* = 0.0900), and alcohol consumption (*p* = 0.6556) between the two groups. Otitis media was more common in the non-BPPV group (*p* = 0.0001; Table 2).

### 3.2 Machine learning based on feature variables

**Figure 1:**
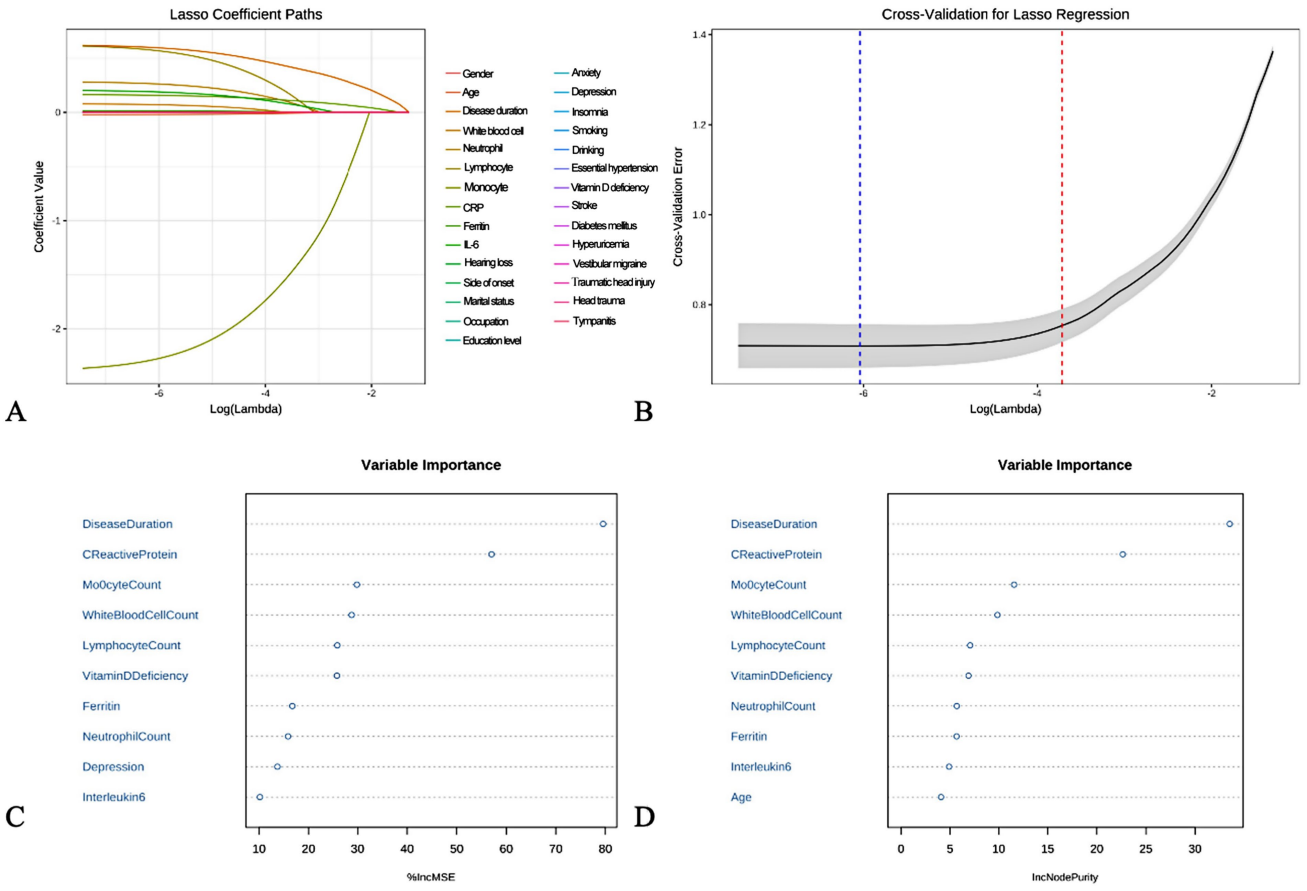
Machine learning based on feature variables. **(A)** LASSO regression analysis. **(B)** LASSO regression screening of targets. **(C,D)** Random forest.

**Figure 2:**
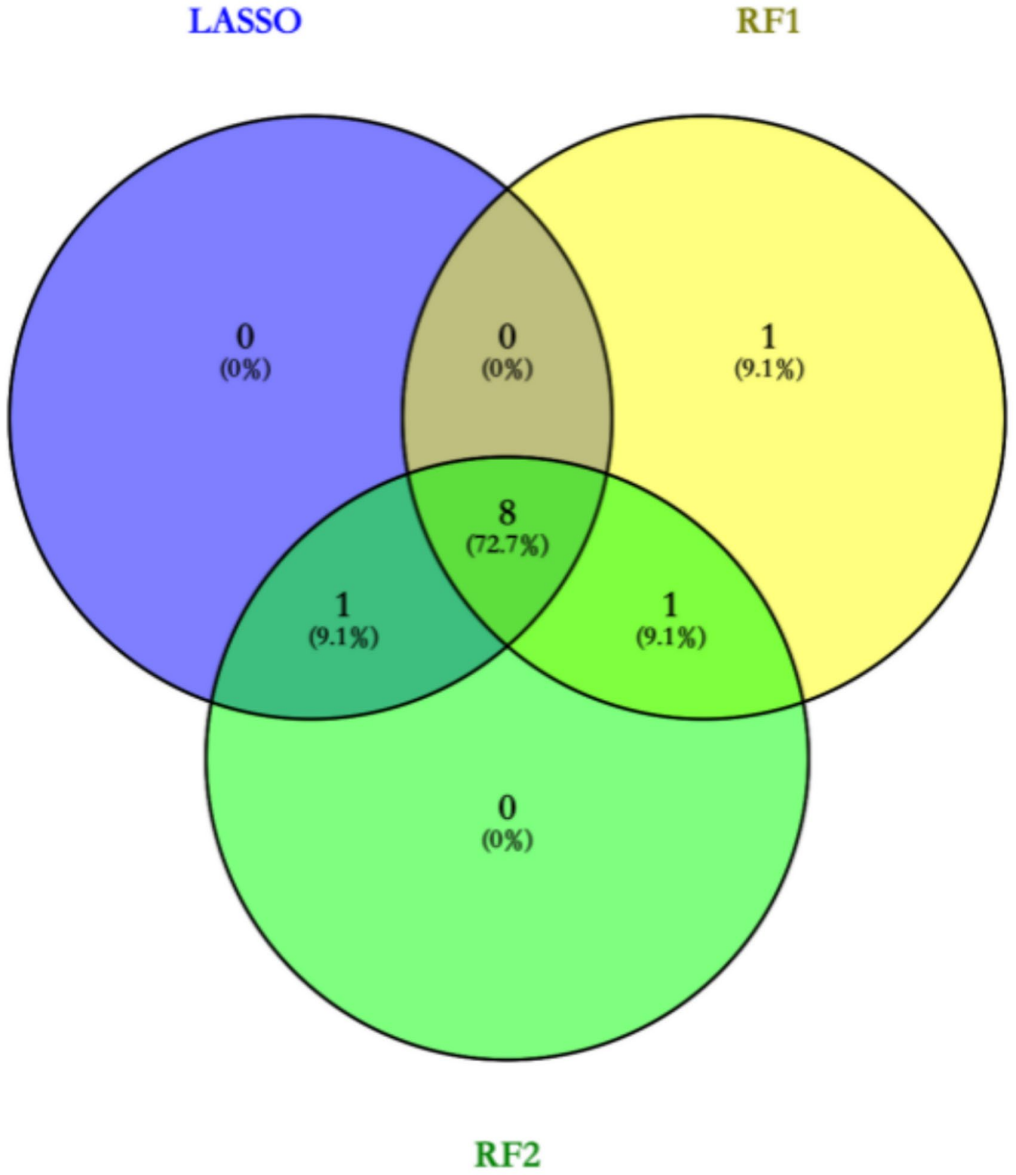
Venn diagram to obtain core targets.

Based on the LASSO regression analysis, a total of 9 core targets were obtained at Lambda$1se, and random forests were used for variable screening using type1 and type2 to obtain the top 10 variables in the sorting, respectively, (Figure 1). The Venn diagram ultimately acquires eight core targets for machine learning: disease course, leukocytes, neutrophils, lymphocytes, monocytes, CRP, ferritin, and vitamin D deficiency (Figure 2).

### 3.3 Multi-factor regression analysis based on characteristic variables and development of predictive models for assisted diagnosis

**Figure 3:**
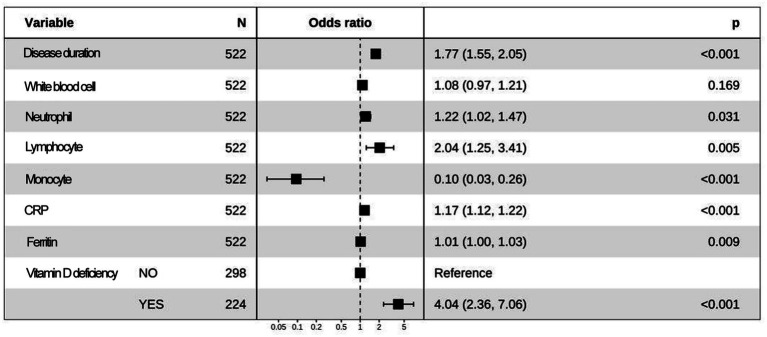
The forest plot shows the results of multivariate logistic regression.

**Figure 4:**
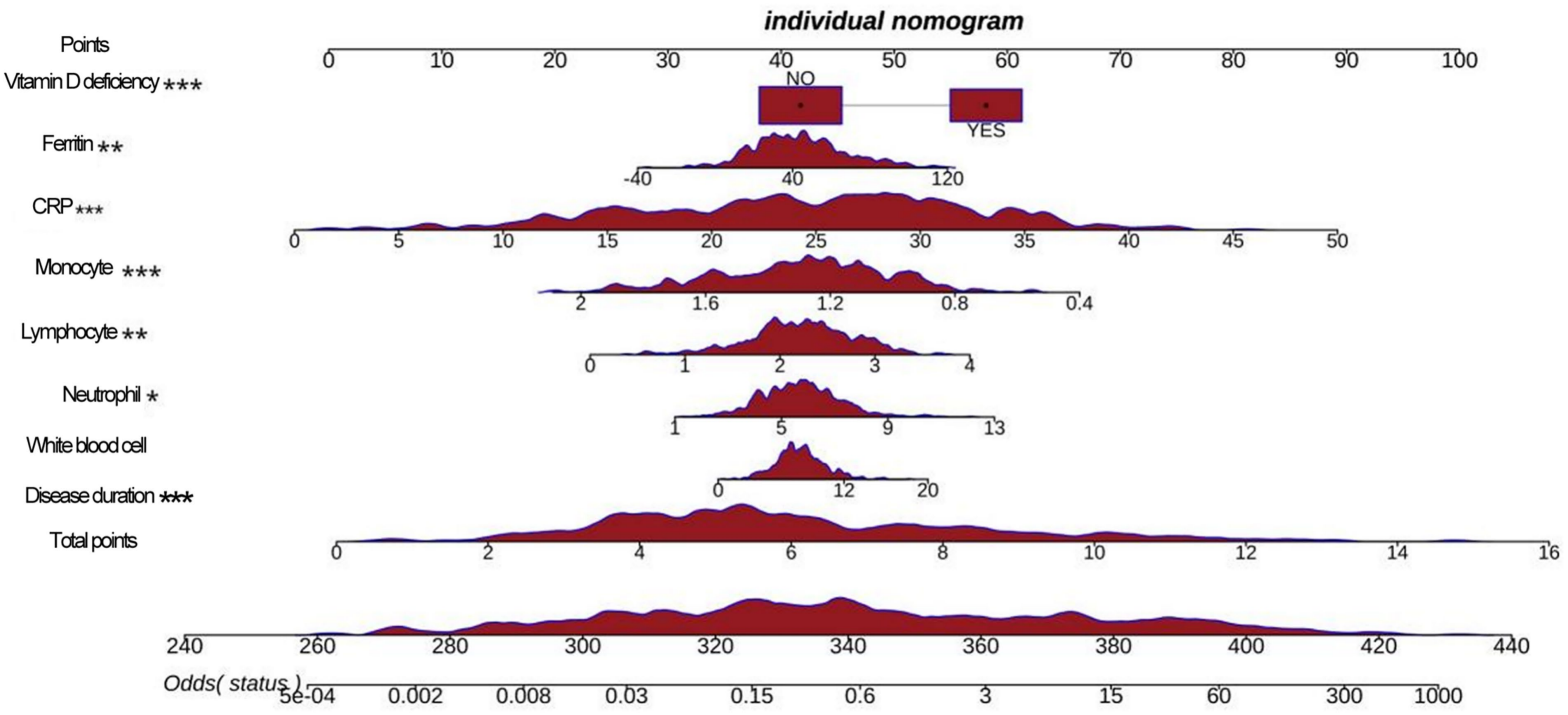
A nomogram shows a predictive model.

Based on machine learning, disease duration, white blood cell, neutrophils, lymphocytes, monocytes, CRP, ferritin, and vitamin D deficiency factors were finally obtained and included in the multifactorial logistic regression analysis (Table 3). The results showed that disease duration, leukocytes, neutrophils, lymphocytes, mono-CRP, ferritin, and vitamin D deficiency, were independent risk factors for the diagnosis of BPPV (OR>1, *p* < 0.05), and monocyte count was an independent protective factor for the diagnosis of BPPV (OR<1, *p* < 0.05). Based on multifactor logistic regression analysis, a prediction model was established as shown in Figures 3, 4.

**Table 3:** Multifactor logistic regression analyses based on the acquired characteristic variables.

Characteristic variables	Estimate	Std. Error	z.value	Pr(>|z|)	OR
Disease duration	0.57	0.07	7.92	<0.01	1.77
White blood cell	0.08	0.06	1.38	0.17	1.08
Neutrophil	0.20	0.09	2.15	0.03	1.22
Lymphocyte	0.71	0.26	2.79	0.01	2.04
Monocyte	(2.35)	0.53	(4.42)	<0.01	0.10
CRP	0.16	0.02	7.20	<0.01	1.17
Ferritin	0.01	0.01	2.62	0.01	1.01
Vitamin D deficiency	1.40	0.28	5.01	<0.01	4.04

### 3.4 External validation based on the auxiliary diagnostic prediction models

**Figure 5:**
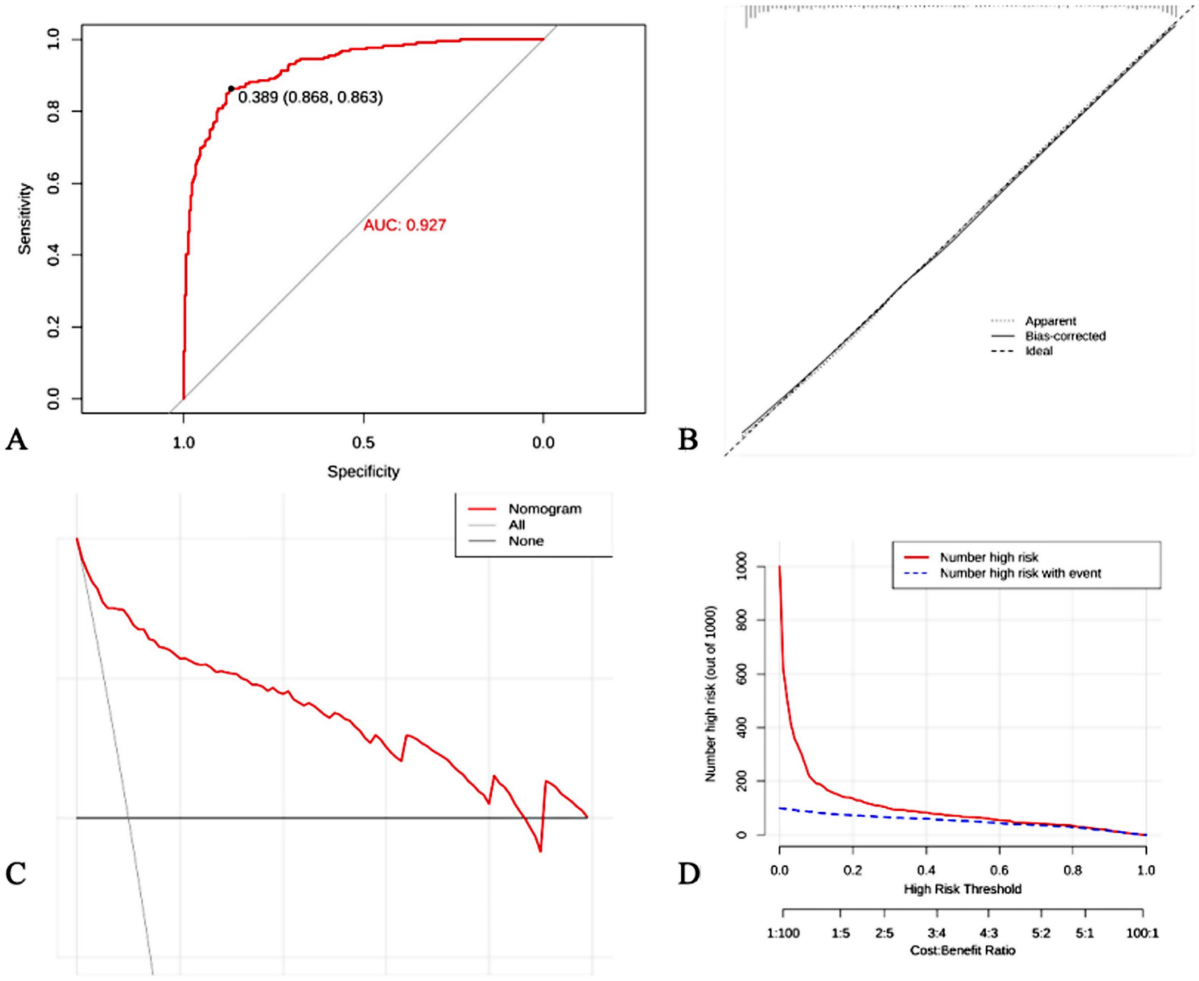
External validation of predictive models. **(A)** ROC curve; **(B)** Clinical calibration curves; **(C)** Clinical decision curves; **(D)** Clinical impact curves.

The established prediction model was externally applied to the patients with BPPV diagnosed by the SRM-IV vertigo diagnosis and treatment system, and the ROC curve was plotted and the AUC was calculated to be 0.927. Further plotting of clinical calibration curves showed that the predicted values were in good agreement with the actual values. Finally, the clinical decision curve and clinical impact curve were plotted, and the established prediction model had a good clinical net benefit rate (Figure 5).

## 4 Discussion

### 4.1 Baseline data of BPPV versus non-BPPV patients were analyzed

In this study, 522 patients with vertigo were included, of which 303 were diagnosed with BPPV and 219 with non-BPPV. Among the 303 BPPV patients, about 76% were posterior canals, 21% were horizontal canals, and 3% were superior canals. The rate of nystagmus disappearance after reduction was 92.7% (281/303). There was no significant difference between the two groups in terms of gender composition, but there was a significant difference in age distribution and duration of the disease. The mean age of the patients in the BPPV group was higher than that of the non-BPPV group. It has been shown that the incidence of BPPV increases with age, which may be related to the deterioration of vestibular function in the elderly (12). In addition, this study found that patients in the BPPV group had a shorter duration of illness than those in the non-BPPV group, suggesting that BPPV may be characterized by a more acute onset.

In terms of laboratory indices, there were significant differences between the two groups in several indices. Patients in the BPPV group had higher levels of monocytes than those in the non-BPPV group, whereas CRP, ferritin and IL-6 levels were lower. These results suggest that inflammatory response and iron metabolism disorders may play an important role in the pathogenesis of BPPV. It has been found that inflammation of the vestibular system can lead to otolith dislodgement and trigger vertigo symptoms (13). Iron metabolism disorders have also been reported to be associated with vestibular dysfunction (14).

In terms of comorbidities, patients in the non-BPPV group had a higher incidence of anxiety and depression than the BPPV group, and vitamin D deficiency was also more common. This suggests that psychological factors and vitamin D levels may be important clues in the differential diagnosis of BPPV (15, 16). It has been shown that mood disorders such as anxiety and depression can cause vestibular dysfunction leading to vertigo symptoms (17, 18). Vitamin D deficiency has also been found to be associated with impaired vestibular function (19). In addition, stroke, diabetes mellitus, hyperuricaemia and history of head surgery were more common in the non-BPPV group, suggesting that these factors may be potential causes of non-BPPV vertigo.

### 4.2 Machine learning based screening of core features for BPPV diagnosis

In this study, two machine learning methods, LASSO regression and random forest, were used to screen out feature variables that are valuable for BPPV diagnosis. LASSO regression obtained 9 core targets at Lambda$1se, while random forest obtained the top 10 ranked variables, respectively. Integration of the results of the two methods through Venn diagrams resulted in the identification of eight core features, including disease duration, white blood cell count, neutrophil count, lymphocyte count, monocyte count, CRP, ferritin, and vitamin D deficiency.

These results are consistent with the findings of the aforementioned baseline data analysis, further confirming the important roles of inflammatory response, iron metabolism disorders and vitamin D deficiency in the pathogenesis of BPPV. Meanwhile, machine learning methods have also revealed the value of clinical indicators such as disease duration and blood cell counts in the diagnosis of BPPV. One study used decision trees and random forests to construct a diagnostic model for vertigo diseases, and found that factors such as age, gender, and the nature of vertigo contributed most to the discriminative power of the model (5). Another study applied support vector machines and artificial neural networks to analyze the results of vestibular function tests to achieve automatic classification and diagnosis of vertigo diseases (20). These studies show that machine learning is expected to uncover key information for diagnosing vertigo diseases from massive clinical data and provide powerful support for clinical decision-making.

### 4.3 Construction of an auxiliary diagnostic model for BPPV based on multifactorial regression analysis

On the basis of the eight core features screened by machine learning, this study further used multifactorial logistic regression analysis to construct an auxiliary diagnostic model for BPPV. The results of regression analysis showed that disease duration, white blood cell count, neutrophil count, lymphocyte count, CRP, ferritin and vitamin D deficiency were independent risk factors for the diagnosis of BPPV, while monocyte count was an independent protective factor. These findings are consistent with the previous discussion, suggesting that inflammatory responses, disturbances in iron metabolism and vitamin D deficiency may be involved in the pathogenesis of BPPV by affecting vestibular system function.

Based on the logistic regression model, this study drew column-line graphs to visualize the relationship between factors and the risk of BPPV, which provides a convenient tool for clinicians to estimate the probability of developing BPPV based on the patient’s specific situation. One study used multifactorial logistic regression to construct a prediction model for BPPV and found that age, duration of vertigo, vertigo triggers and vestibular function test results were independent predictors of BPPV (21). This is partially similar to the findings of this study, suggesting that clinical characteristics and vestibular function indicators are valuable for the diagnosis and prediction of vertigo disorders.

### 4.4 External validation and clinical application value of a companion diagnostic model

In order to assess the actual performance of the constructed auxiliary diagnostic model, external validation was performed in this study using data from patients with BPPV diagnosed by the SRM-IV Vertigo Diagnostic and Treatment System. ROC curve analysis showed that the model had a high predictive accuracy for BPPV, with an AUC of 0.927. Calibration curve analysis also confirmed the good agreement between the model’s predicted probability and the actual incidence rate. In addition, decision curve and clinical impact curve analyses showed that the model could provide a net benefit for clinical decision making and had good clinical application value.

These results support the usefulness of the auxiliary diagnostic model constructed in this study in the diagnosis of BPPV. By incorporating multidimensional information such as clinical features and laboratory indicators, the model is able to provide an objective and quantitative basis for the diagnosis of BPPV, which helps to improve the accuracy and efficiency of diagnosis. Similarly, one study used machine learning to construct a prediction model for BPPV and validated it on an external dataset, confirming the reliability and usefulness of the model (22, 23). Another study integrated a machine-learning diagnostic model for vertigo disease into a clinical decision support system and achieved good application results (24). These studies show that the auxiliary diagnostic models are expected to provide intelligent tools for the diagnosis and treatment of vertigo diseases, reduce the workload of doctors, and improve the level of diagnosis and treatment.

However, this study was a retrospective single-center design with selection bias. Potential variables such as serum calcium/bone metabolism indexes were not included; there were differences in the time intervals between laboratory tests and symptom onset; and the model did not incorporate data on quantitative assessment of vestibular function and response to treatment, which limited its value for clinical application. Based on the findings of this study, future research should conduct a multicenter prospective study to validate the external validity of the model; in addition, the relationship between the dynamic changes in biomarkers and the prognosis of BPPV should be explored; and clinical, laboratory, and imaging data should be integrated to construct a multimodal prediction model. In-depth mechanistic studies should be conducted to elucidate the molecular pathways of inflammation and metabolic disorders leading to BPPV.

## 5 Conclusion

In this study, the clinical characteristics of BPPV and non-BPPV patients were compared and analyzed, and the core diagnostic indicators were screened by machine learning methods, and the auxiliary diagnosis and prediction model of BPPV was constructed and externally verified. The findings provide new evidence for the pathophysiologic mechanisms of BPPV, suggesting that inflammatory responses and metabolic disorders may be involved in the pathogenesis of BPPV. However, due to the retrospective and single-center design of this study, these association findings need to be further validated by multicenter prospective studies.

## Data Availability

The original contributions presented in the study are included in the article/supplementary material, further inquiries can be directed to the corresponding author/s.

## References

[1] Choi HG Kim G Kim BJ Hong SK Kim HJ Lee HJ. How rare is benign paroxysmal positional vertigo in children? A review of 20 cases and their epidemiology. Int J Pediatr Otorhinolaryngol. (2020) 132:110008. doi: 10.1016/j.ijporl.2020.110008, PMID: 32240880

[2] Guo T Jia G Liu D Deng X Li J Xie H. Understanding factors that cause benign paroxysmal positional Vertigo, Ménière disease, and vestibular neuritis: a two-sample Mendelian randomization study. Ear Hear. (2025) 46:305–14. doi: 10.1097/AUD.0000000000001574, PMID: 39145629

[3] Hannan MA Barman K Begum A. Epidemiological and clinical study of vertigo in a tertiary care hospital of Bangladesh. J Adv Med Med Res. (2022) 1:54–60. doi: 10.9734/JAMMR/2022/v34i2231578

[4] Fangzhou Y Peixia W Haowen D. A questionnaire-based ensemble learning model to predict the diagnosis of vertigo: model development and validation study. J Med Internet Res. (2022) 24:e34126. doi: 10.2196/34126, PMID: 35921135 PMC9386585

[5] Tang X Ye W Ou Y Ye H Zhu X Huang D. Development and validation of a machine learning model for detection and classification of Vertigo. Laryngoscope. (2025) 135:1652–60. doi: 10.1002/lary.31959, PMID: 39698985

[6] Soylemez E Demir S Ozacar K. Machine learning-based Mobile application for predicting Posterior Canal benign paroxysmal positional Vertigo. Laryngoscope Investig Otolaryngol. (2025) 10:e70177. doi: 10.1002/lio2.70177, PMID: 40521132 PMC12166311

[7] Baydan-Aran M Binay-Bolat K Söylemez E Aran OT. Predictive modeling of maneuver numbers in BPPV therapy using machine learning. J Vestib Res. (2025) 13:1905. doi: 10.1177/09574271251351905, PMID: 40512135

[8] Mun SB Kim YJ Lee JH. Deep learning-based nystagmus detection for BPPV diagnosis. Sensors (Basel). (2024) 24:417. doi: 10.3390/s24113417, PMID: 38894208 PMC11175138

[9] Xing J Yang P Yun Y Cheng Z Han P Zhang T. Discussion and analysis the value of supine median^3^ nystagmus in the diagnosis and treatment of HC-BPPV. Lin Chung Er Bi Yan Hou Tou Jing Wai Ke Za Zhi. (2024) 38:432-435;441. doi: 10.13201/j.issn.2096-7993.2024.05.016, PMID: 38686483 PMC11387321

[10] Yang J Xiong G Lu H Luo X Xie X Shao A. Residual dizziness characteristics of idiopathic sudden sensorineural hearing loss patients with benign paroxysmal positional vertigo. Audiol Neurootol. (2024) 30:45–51. doi: 10.1159/000540036, PMID: 39068920 PMC11809453

[11] Yu J Gu Y Meng G Zhu X Wang W Liu X. Nystagmus parameters of supine roll test correlates with prognosis after repositioning maneuver in horizontal Semicircular Canal benign paroxysmal positional Vertigo. Front Neurol. (2021) 12:790430. doi: 10.3389/fneur.2021.790430, PMID: 34938267 PMC8687044

[12] Tomaz A Ganança MM Ganança CF Ganança FF Caovilla HH Harker L. Benign paroxysmal positional vertigo: concomitant involvement of different semicircular canals. Ann Otol Rhinol Laryngol. (2009) 118:113–7. doi: 10.1177/000348940911800206, PMID: 19326761

[13] Hu Y Lu Y Wang S Quan X Ren Y Rong K. Global research trends in benign paroxysmal positional vertigo: a bibliometric analysis. Front Neurol. (2023) 2:1204038. doi: 10.3389/fneur.2023.1204038, PMID: 37333008 PMC10272773

[14] Zhou F Fu M Zhang N Xu Y Ge Y. Investigation of the relationship between chronic diseases and residual symptoms of benign paroxysmal positional vertigo. Lin Chung Er Bi Yan Hou Tou Jing Wai Ke Za Zhi. (2015) 29:1627–9. PMID: 26790263

[15] Arshad Q Cousins S Golding JF Bronstein AM. Factors influencing clinical outcome in vestibular neuritis - a focussed review and reanalysis of prospective data. J Neurol Sci. (2023) 446:120579. doi: 10.1016/j.jns.2023.120579, PMID: 36807973

[16] Lahiji MR Akbarpour M Soleimani R Asli RH Leyli EK Saberi A. Prevalence of anxiety and depression in Meniere's disease; a comparative analytical study. Am J Otolaryngol. (2022) 43:103565. doi: 10.1016/j.amjoto.2022.103565, PMID: 35981431

[17] Jiang CY Wu J Shu L Sun XH Pan H Xu Q. Clinical and cVEMP evaluation predict short-term residual dizziness after successful repositioning in benign paroxysmal positional Vertigo. Front Med. (2022) 9:881307. doi: 10.3389/fmed.2022.881307, PMID: 35685419 PMC9170995

[18] Yan S Li Z Chen P Wu W. Influencing factors for residual symptoms following canalith repositioning maneuver in patients with benign paroxysmal positional vertigo. Ear Nose Throat J. (2025) 19:4913. doi: 10.1177/01455613241304913 39829109

[19] Zhang ZY Tian SF Li H Cao XW Song Y. The correlations between serum vitamin D, parathyroid hormone, and bone mineral density with benign paroxysmal positional vertigo. Lin Chung Er Bi Yan Hou Tou Jing Wai Ke Za Zhi. (2019) 33:504–7. doi: 10.13201/j.issn.1001-1781.2019.06.007, PMID: 31163522

[20] Xu X Jiang R Zheng S Wang M Ju Y Li J. Classification of chronic dizziness using large language models. J Healthc Inform Res. (2024) 9:88–102. doi: 10.1007/s41666-024-00178-1, PMID: 39897102 PMC11782766

[21] Rastall DP Green K. Deep learning in acute vertigo diagnosis. J Neurol Sci. (2022) 443:120454. doi: 10.1016/j.jns.2022.120454, PMID: 36379134

[22] Halmágyi GM Akdal G Welgampola MS Wang C. Neurological update: neuro-otology 2023. J Neurol. (2023) 270:6170–92. doi: 10.1007/s00415-023-11922-9, PMID: 37592138 PMC10632253

[23] Mamykin GD Kulesh AA Barkov FL. Methods for detecting the patient’s pupils’ coordinates and head rotation angle for the video head impulse test (vHIT), applicable for the diagnosis of vestibular neuritis and pre-stroke conditions. Computation. (2024) 12:167. doi: 10.3390/computation12080167

[24] Suresh K Elkahwagi MA Garcia A Naples JG Corrales CE Crowson MG. Development of a predictive model for persistent dizziness following vestibular schwannoma surgery. Laryngoscope. (2023) 133:3534–9. doi: 10.1002/lary.30708, PMID: 37092316 PMC10593906

